# Visual Search Performance Does Not Relate to Autistic Traits in the General Population

**DOI:** 10.1007/s10803-019-03907-3

**Published:** 2019-02-16

**Authors:** David López Pérez, Daniel P. Kennedy, Przemysław Tomalski, Sven Bölte, Brian D’Onofrio, Terje Falck-Ytter

**Affiliations:** 1 Neurocognitive Development Lab, Faculty of Psychology, University of Warsaw, 5/7, 00-183 Warsaw, Poland; 2 Department of Psychological and Brain Sciences, Indiana University, Bloomington, IN USA; 3 Uppsala Child & Babylab, Department of Psychology, Uppsala University, Box 256, 751 05 Uppsala, Sweden; 4 Center of Neurodevelopmental Disorders at Karolinska Institutet (KIND), Neuropsychiatry Division, Department of Women’s and Children’s Health, Karolinska Institutet, 171 77 Stockholm, Sweden; 5 Child and Adolescent Psychiatry, Center for Psychiatry Research, Stockholm County Council, 11330 Stockholm, Sweden; 6 Swedish Collegium for Advanced Study (SCAS), Uppsala, Sweden

**Keywords:** Visual search task, Autism spectrum disorder, Eye tracking, Reaction times, Eye movements

## Abstract

Autism spectrum disorder (ASD) is commonly conceived as the extreme end of a continuum. Research suggests that autistic individuals outperform typically developing controls in visual search. Thus, enhanced visual search may represent an adaptive trait associated with ASD. Here, using a large general population sample (N = 608, aged 9–14 years), we tested if higher levels of autistic traits are associated with enhanced visual search. Visual search was evaluated using both manual responses and eye movements, and autistic traits were measured using the Social Responsiveness Scale. Contrary to our hypothesis, no significant relation between autistic traits and visual search were observed. The theoretical implications of these results are discussed.

**Electronic supplementary material:**

The online version of this article (10.1007/s10803-019-03907-3) contains supplementary material, which is available to authorized users.

## Introduction

In everyday life, one often has to search for visual targets among distractors, for example in order to find a specific item in the fridge or wardrobe. Effective visual search can be a rather demanding cognitive task, particularly if the target and the background share many characteristics (e.g., Hershler and Hochstein 2005). On the other hand, sometimes visual targets are salient from the background and take no or minimal mental effort to detect. Because these visual processes directly constrain and guide our behaviour, visual search has been extensively studied in research on humans as well as animals (e.g., Land 1999; Lobue and Deloache 2008).

Autism Spectrum Disorder (ASD) is a heritable and relatively common condition defined by impairments in social interaction and communication, as well as restricted and repetitive behaviours and interests. Previous research suggests that visual search is an area of strength in ASD. For example, Plaisted et al. (1998) found that 7- to 10-year-olds with ASD were faster than controls at detecting conjunctive targets—that is, targets sharing one feature with two different distractor types (e.g., orange horizontal bar among several blue horizontal and orange vertical bars). O’Riordan et al. (2001) found that 9-year-olds with ASD identified the target faster than controls for difficult searches, defined as either feature search (in which one stimulus characteristic is enough to identify a target) with large set size or as conjunctive search. In other words, difficulty did not affect reaction times in ASD as much as in controls. This ASD advantage was found for both target present and target absent trials. Summarizing a range of studies in this area over the last 15 years, Kaldy et al. (2016) concluded that the visual search performance advantage in ASD is a robust finding. Additionally, research indicates that the ASD advantage in visual search may have an early onset, starting as early as in infancy (Gliga et al. 2015). While efficient visual search in itself is adaptive, it may come at a cost of a detailed-focused processing style that may be sub-optimal in other areas, such as perception of complex social information (e.g., Nilsson Jobs et al. 2018).

ASD is today commonly operationalized as the extreme end of a phenotypic continuum of autistic traits normally distributed in the general population (Lundström et al. 2012; Robinson et al. 2015). From this perspective, it is expected that phenotypes linked to ASD, such as enhanced visual search ability, should also be found in individuals with high autistic traits even without an autism diagnosis.

A few of studies have previously investigated visual search in relation to autistic traits in the general population (e.g., Brock et al. 2011; Gregory and Plaisted-Grant 2016). These studies generated mixed results. For example, Gregory and Plaisted-Grant (2016) found no association between scores on the Autism Quotient (AQ) and several visual search measures. However, all prior studies have suffered from rather limited sample size (N < 100), which renders low power as a possible explanation for previous negative results. Therefore, we recruited a substantially larger (N = 608) sample of school-age children. Further, in contrast to the earlier studies, we analysed both their manual responses (key press, indicating whether a target was present or not) and eye movements during the task (e.g., how long it took them to fixate the targets). Based on the work with individuals with a diagnosis of ASD, we predicted that visual search performance would be positively correlated with the level of autistic traits, as quantified using the Social Responsiveness Scale (SRS; Constantino and Gruber 2005). Specially, we focused on the analysis of reaction times, where an ASD advantage has been extensively reported during visual search tasks (Kaldy et al. 2016). By obtaining both manual and eye movement responses, we could also explore the relation between autistic traits and other several other measures of potential interest, such as latency to fixate the target object. We expected the reaction times during difficult trials (i.e., conjunctive at larger set sizes) to be negatively associated with autistic traits, and that high autistic traits would be associated with being less affected by set size for these difficult searches.

## Methods

### Participants

Participants were 9–14 year old children recruited from The Child and Adolescent Twin Study in Sweden (CATSS), a longitudinal study of twins born in Sweden (Anckarsäter et al. 2011). About 70% of all twins in Sweden are included in the CATSS study. From this larger study, the current study recruited a subsample of monozygotic and same sex dizygotic twins living in the Stockholm area. Eleven children were tested, but subsequently excluded because uncorrected problems of vision or hearing, serious medical conditions (e.g., epilepsy) or genetic syndromes e.g., TSC, FXS, 22q11, 16p11.2, Rett’s syndrome (general exclusion criteria). We did not pursue any twin analyses in the current study.

Eye-tracking data was collected from 723 children, but after quality control procedures (see data processing for more details) only 608 were included in the final analysis. No differences in age, IQ or SRS were found between those participants included in the analysis and those excluded (see Supplementary Methods). Written informed consent was obtained from the parents of all participants. The study was approved by the regional ethical committee in Stockholm, Sweden, and was conducted in accordance with the Declaration of Helsinki.

Parents completed the Social Responsiveness Scale (Constantino and Gruber 2005), a common measure of autistic traits (raw scores used in analyses). As expected, in our large sample from the general population we observed a large spread and a somewhat positively skewed distribution (histograms shown in Supplementary Figure 3). As recommended for research settings, we used raw scores for the main analyses in this article, but results remained essentially unchanged if we used T-scores instead (see Supplementary Table 1). Four subscales (vocabulary, digit span, matrices, coding) from the Wechsler Intelligence Scale for Children—4th Edition (WISC-IV; Kaufman et al. 2006) were administered to the children individually by a psychologist during the visit. The average of the standard scores for these WISC-IV subtests was used in the main analyses (see Supplementary Table 1 for analysis of the individual subscales).

### Procedure

Stimuli were presented on a 23″ monitor with a resolution of 1024 × 1280 pixels and responses were registered using a USB keyboard. The CIE coordinates of the stimuli were [0.26, 0.23, 0.98] for blue, [0.49, 0.36, 0.04] for orange and [0.95, 1, 1] for white background. The visual angle of the monitor was 29.32° if width and 24.22° of height. The average luminance was 188 cd/m^2^. The experiment was performed using a Tobii T120 eye tracker recording at a 120 Hz sampling rate. Participants completed 10 training trials, during which they were trained to hold their index fingers stable on separate keys during the whole experiment, in order to be able to perform the task without looking at the keyboard. The experimenter did not start the session until they were able to do this effortlessly. Subsequently, a 9-point calibration image was used to determine the positions of the eyes before the task began. The task begun only after a successful calibration was achieved according to the experimenter (procedure repeated if necessary). Each participant completed a total number of 120 trials presented in a random order, separated by two short breaks.

The visual search task used rectangular objects that varied in colour and orientation (see Supplementary Fig. 2 for example). The participants were instructed to determine if one unique target was present in each array, and press with their left index finger if a target was present and with their right index finger if the target was absent. During the 10 training trials, the experimenter ensured that they understood these instructions and were able to follow them for all conditions (see below). They were instructed to press as soon as they could.

The experiment consisted of 12 unique conditions, produced by manipulation of 3 independent variables, as follows. First, we had two types of search—both feature and conjunction search. In the feature search, an array of items is shown in which a ‘target’ item (the item that is to be searched for) has a unique feature that distinguishes it from a homogeneous set of distractors (e.g., an orange rectangular ‘target’ in a field of blue rectangular distractors). In the conjunction search task, the target item had a variation of two different feature dimensions (e.g., an orange horizontal rectangle among a distractor set containing both orange and blue rectangles placed horizontally and vertically). The ‘target’ item was defined by its uniqueness relative to the distractors on a trial to trial basis (i.e., unless it happened by chance, the target was typically different in each trial - orange horizontal rectangle in one trial and then blue vertical target in another). Second, we varied the number of elements in the task and used three different set sizes (i.e., 8, 18 and 28 elements). Finally, the ‘target’ item could be present or absent in the task. These manipulations were selected based on the prior work on visual search in individuals with ASD (e.g., Plaisted et al. 1998; O’Riordan et al. 2001). The number of trials were fully balanced across conditions. The trial ended when the participant pressed the key, but the experimenter was instructed to prompt the child to press if he or she did not respond within about 10 s (this very rarely happened).

### Data Processing

Data was pre-processed using in-house scripts written in Matlab 2017a (The MathWorks Inc., Natick, MA, 2017). First, trials were included in the analysis if they contained at least 70% of valid gaze samples for both eyes as defined by the Tobii eye tracker output. Given that the eye tracker quickly loses track of the eyes if one moves one’s gaze away from the screen and that it was surrounded by a plain black background, valid gaze data will almost exclusively reflect moments when the participant actually looked at the screen. Next, reaction times (RTs) were calculated from the onset of the stimuli until a recorded manual key press (i.e., “z” for target present and “m” for target absent). A key-press was considered valid if contained one of the instructed keys or immediately surrounding keys (to allow for possible trivial mistakes in key-pressing). If any other key was pressed, the trial was considered invalid. Based on visual inspection of the data and assuming that unreasonably fast RTs represented anticipations and unreasonably slow RTs represented attentional lapses, we considered invalid all trials with RTs < 300 ms or > 7000 ms for the feature task and RTs < 300 ms or > 10,000 ms for conjunction search tasks. A total of 8.97% of the entire data set for all observers across all tasks was removed by this method. Given the large sample size and the relatively low % of excluded data, we can expect that truncation of the RT data set in this manner will have little or no effect on the results (this was also supported by sensitivity analyses, see Supplementary Table 6). Mean RT values were used for the analysis because visual inspection of within-individual data did not show any obvious violation of normality. Additionally, visual search efficiency was calculated by comparing slopes and intercepts for the set-size by RT function (e.g., O’Riordan et al. 2001).

Fifty-six participants were excluded for further analysis due to experimental errors (e.g., disruptions during experiment), 27 because the ET files were corrupted, and 21 because they did not provide at least 50% valid trials. In this report, we primarily focused on the analysis of RTs but, as noted above, other metrics were also investigated (further details regarding the data analysis is available in the supplementary information).

## Results

### Visual Search in Relation to Sex, Age and IQ

**Fig. 1:**
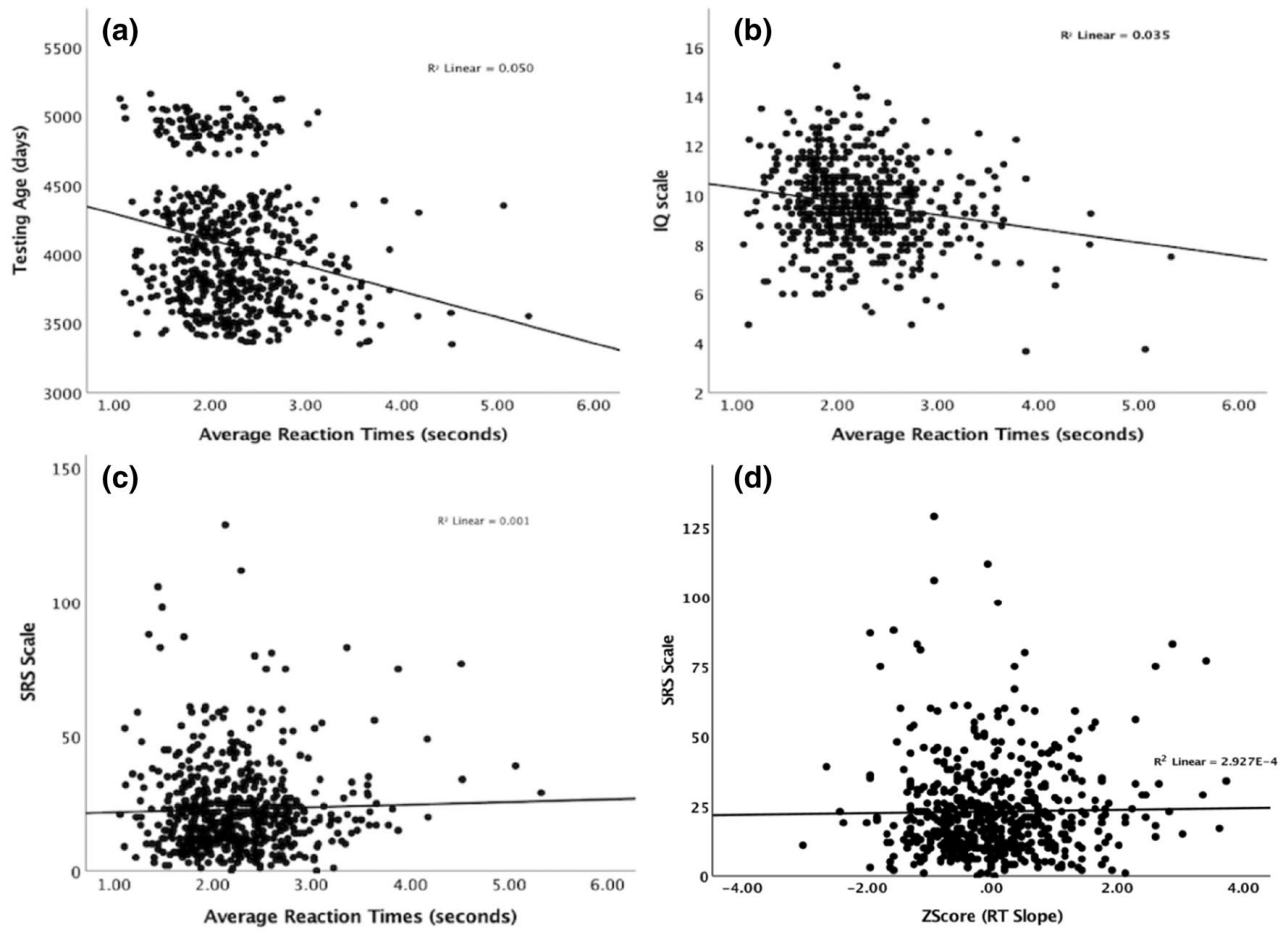
Linear regressions between the average RTs (across all conditions) and age (**a**), the average IQ scale (four subscales from the WISC-IV) (**b**), the SRS scale (**c**). **d** Shows the relation between visual search efficiency (slope of the set size by reaction time function) for all conditions and the SRS scale

We did not find any sex differences (that survived correction for multiple testing; Supplementary Table 1) and therefore we did not control for it in subsequent analysis. We found significant negative correlations between RTs and age, with older children performing faster in all conditions (Fig. 1a and Supplementary Table 1). As shown in Fig. 1b (see also Supplementary Table 1), we also found significant negative correlations between RTs and IQ, wherein participants with higher IQ had shorter reaction times. In the correlations shown below we did not control for any of the aforementioned effects (e.g., age or IQ), since additional tests controlling for these variables did not affect the results (see Supplementary Table 6).

### Visual Search in Relation to Autistic Traits

We did not observe any statistically significant correlations between visual search (RTs) and autistic traits (SRS scores) neither for the condition average (r_average_ (587) = 0.03, *p* = .43) nor for any of the conditions, including the most difficult trials, such as the largest set size in conjunctive search (r_28_absent_(584) = 0.00, *p* = .90; r_28_present_(584) = 0.00, *p* = .90; see also Fig. 1c and Supplementary Tables 1 for data for remaining conditions). Similarly, we did not find any statistically significant relation between visual search efficiency (intercept and slope of set size by reaction time function) and SRS scores for any condition (Fig. 1d; Table 1). Additionally, no statistically significant correlations were found for the visual search measures derived from eye movements (i.e., time to first fixation at target, and gaze fixation vs. keypress-difference), as well as for the accuracy (i.e. correctly indicating whether a target was present or not; see Supplementary Table 2). Likewise, no statistically significant relations were found between the visual search efficiency of these measures and autistic traits (see Supplementary Table 3).

**Table 1:** Correlations between the reaction times visual search efficiency (intercept and slope of the set size by reaction time function) and SRS scales, and between reaction times and SRS scales

	All conditions	Present	Absent	Conjunction	Feature
RT slope	r(579) = 0.02	r(577) = − 0.02	r(573) = − 0.02	r(576) = − 0.02	r(575) = − 0.02
	*p* = .63	*p* = .50	*p* = .51	*p* = .60	*p* = .58
RT intercept	r(579) = 0.01	r(577) = 0.01	r(573) = 0.03	r(576) = 0.02	r(575) = 0.01
	*p* = .71	*p* = .66	*p* = .42	*p* = .56	*p* = .77
RTs	r(587) = 0.03	r(584) = 0.01	r(584) = 0.03	r(583) = 0.01	r(584) = 0.04
	p = .43	p = .72	p = .40	p = .68	p = .27

The n varies slightly between analyses due to the availability of the different measures and the removal of outliers

### Extreme Group Analyses

**Fig. 2:**
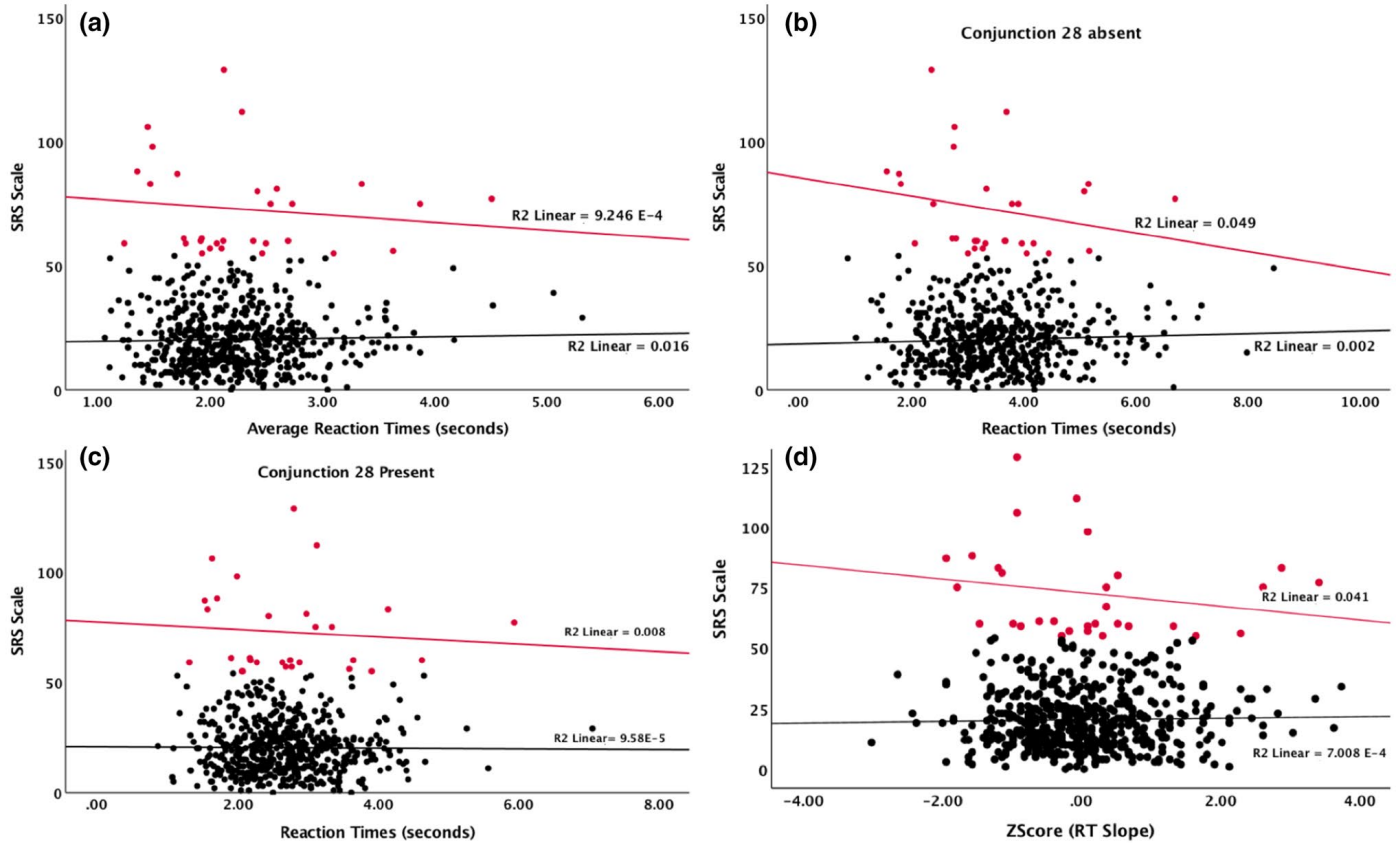
Linear regressions between SRS scores (in red SRS scores ≥ 55 and in black SRS scores < 55) and RTs for the grand average (**a**), the conjunction 28 absent (**b**) and conjunction 28 present (**c**) conditions, and the between the visual search slope for the all conditions average (**d**)

To examine if an association might be limited to participants with extreme autistic traits, we formed SRS extreme groups—one low-to-average range (SRS < 55) and one high range group (SRS ≥ 55, corresponding to about 5% of the sample or 30 participants). We ran the above correlations separately for each group. Again, we did not find any statistically significant correlation in any of the groups for any of the conditions (SRS ≥ 55: r_average_ (30) = − 0.12, *p* = .51; r_28_absent_ (587) = − 0.22, *p* = .24; r_28_present_ (587) = − 0.08, *p* = .65; SRS < 55: r_average_ (557) = 0.03, *p* = .43; r_28_absent_ (554) = 0.04, *p* = .43; r_28_present_ (555) = − 0.01, *p* = .43; see also Fig. 2a-c and Table 2 and Supplementary Table 4 for the remaining conditions). Similarly, we did not find any statistically significant relation between visual search efficiency (intercept and slope) measures and SRS scores for any condition (SRS ≥ 55: r_intercept_ (30) = − 0.12, *p* = .51; r_slope_ (587) = − 0.22, *p* = .24; SRS < 55: r_intercept_ (557) = 0.03, *p* = .43; r_slope_ (554) = 0.04, *p* = .43; see also Fig. 2d and Table 2 and data for the remaining conditions can be found in Supplementary Table 5).

**Table 2:** Correlations between RTs and SRS scales and between SRS and visual search efficiency (intercept and slope of the set size by reaction time function) for participants with SRS ≥ 55 and for participants with SRS < 55

	Conditions average	Conjunction 28 absent	Conjunction 28 present	All conditions intercept	All conditions slope
SRS ≥ 55	r(30) = − 0.12	r(29) = − 0.22	r(30) = − 0.08	r(30) = 0.00	r(30) = − 0.20
	*p* = .51	*p* = .24	*p* = .65	*p* = .98	*p* = .28
SRS < 55	r(557) = 0.03	r(554) = 0.04	r(555) = − 0.01	r(552) = − 0.01	r(552) = 0.02
	*p* = .47	*p* = .24	*p* = .81	*p* = .78	*p* = .55

The n varies slightly between analyses due to the availability of the different measures

Next, we computed a Mann Whitney test to compare the RTs between the high range and the low-to average range groups. We did not observe any significant difference, either in the grand average RTs (*U*_*average*_ = 8266.0; *p* = .88, *d* = − 0.00) or for the most difficult trials (*U*_*conj_28_absent*_ = 7795.5, *p* = .61, *d* = − *0.0*2; *U*_*conj_28_present*_ = 8043.0, *p* = .71, *d* = − 0.00) (see Supplementary Table 4 [footnote] for the rest of conditions). Finally, we did not observe any significant difference between visual search efficiency of RTs between the groups averaging across all conditions (*U*_*all_cond_interc*_ = 8529.5; *p* = .66, *d* = 0.16; *U*_*all_cond_slope*_ = 8941.5; *p* = .98, *d* = 0.14) (Supplementary Table 5 [footnote] for results for specific conditions).

## Discussion

Autism spectrum disorder (ASD) is commonly conceived as the extreme end of a phenotypic continuum, encompassing the whole population. This view is supported both by twin analyses (Lundström et al. 2012; Robinson et al. 2011; Colvert et al. 2015) and molecular genetic analyses (Robinson et al. 2015). From this perspective, studies of children from the general population should be of direct relevance to our understanding of the full blown clinical cases and *vice versa*. Based on this assumption, we tested the hypothesis that higher autistic traits in the general population would be associated with better performance in visual search tasks.

Contrary to our prediction, and despite the fact that we examined a large sample of 608 children, we observed no statistically significant correlation between visual search and autistic traits. This contrasts with one earlier study which found that adults with high level of autistic traits outperformed individuals with low level of autistic traits in terms of visual search (Brock et al. 2011), but replicates the hitherto largest previous study of this sort, which found no relation between visual search and scores on the AQ in 97 adults (Gregory and Plaisted-Grant 2016). This negative result observed in our study was obtained using manual reaction times (Fig. 1; Table 1 and Supplementary Table 1)—the measure most consistently linked to ASD previously—and several other visual search measures, including those derived from eye movements (see Supplementary Tables 2 and 3). For example, by computing the difference in time between eye fixation on the target and the manual response, we get a measure of post selection top down processes (i.e. after target is attended, how long does it take to decide if it fulfils the criterion of uniqueness?). Neither for this measure, or the latency of eye movements to targets, we found any strong relation to autistic traits. Sensitivity analyses indicated that the main results were stable across various different parameter settings for data analysis (see Supplementary Table 6). Altogether, this suggests that despite that enhanced visual search may be a robust phenomenon in clinical populations with ASD (O’Riordan et al. 2001; Kaldy et al. 2016), this aspect of the autism appears not to follow a continuous distribution encompassing autistic-like traits in the general population.

One possible explanation for this pattern could be that enhanced visual search is only found in a sub-group of people with ASD, for example those with very high symptom levels. While we had a substantial spread in SRS scores (Fig. 2 and Supplementary Fig. 3) and had a large sample, we were still underpowered to find weak effects at the extreme end of the distribution. Another possibility is that visual search is related to aspects of ASD that may not be covered well by the SRS, such as sensory, attentional or perceptual atypicalities (e.g., Robertson et al. 2013; Pellicano et al. 2013; Kaldy et al. 2016). However, if these traits are strongly linked to ASD, they would also be expected to correlate with the core traits covered by the scale; hence this explanation does not appear very likely. Next, one notable aspect of our study was that the target was defined on a trial-to-trial basis, rather than being predefined and fixed across several trials. Interestingly, using the same approach as the current experiment, Keehn and Joseph (2016) recently found that children with ASD were marginally *worse* than controls in conjunctive search. While we cannot rule out the possibility that this aspect of our experiment is relevant for the result, it is noteworthy that the above-mentioned previous study by Gregory and Plaisted-Grant (2016) on the relation between AQ and visual search in adults without autism failed to find an association despite using predefined and fixed targets. Thus, the two largest studies of visual search in relation to autistic traits so far both failed to find robust relations, despite having applied rather different methodologies. These findings highlight the importance of conducting further large scale case-control studies, and compare clinical cases with ASD with groups of highly elevated autistic traits as well as other clinical groups (e.g., with ADHD). If it turns out that visual search does not show signs of continuity between affected and non-affected individuals even at the more extreme end of the distribution, this is of theoretical and potential practical interest, as this would suggest it being highly specific to ASD caseness (Nilsson Jobs et al. 2018).

While we found no statistically significant relation between visual search performance and autistic traits, we did find several other and rather expected relations. Specifically, overall performance increased with age (9–14 years), and higher average full-scale IQ (and particularly higher scores on the subscale Coding; see Supplementary Tables 1) were associated with faster reaction times during the visual search task.

## Conclusions

Taken together, our behavioural and eye tracking data from 608 children failed to support the hypothesis that higher autistic traits in the general population is associated with better performance in visual search. This finding motivates more research into visual search at the interfaces between extreme autistic traits and full clinical diagnosis.

## Electronic supplementary material

Below is the link to the electronic supplementary material.

Supplementary material 1 (DOCX 20071 KB)
