# Supplementary material for: Visual Search Performance Does Not Relate to Autistic Traits in the General Population
Source: J Autism Dev Disord. 2019 Feb 16;49(6):2624–31. doi: 10.1007/s10803-019-03907-3 (PMC6546657; doi:10.1007/s10803-019-03907-3)
Supplement: Supplementary file 1 — Supplementary material 1 (DOCX 20071 KB) [file 10803_2019_3907_MOESM1_ESM.docx]

Supplementary information for article:

**Brief Report: Visual Search Performance is not Associated with Autistic Traits in the General Population**

***Journal of Autism and Developmental Disorders***

**Supplementary methods**

***Excluded participant vs. Included Participant***

Independent *t-*test showed that included and excluded participants did not differ in age (*t*(721) = 1.16, *p = .24, d = .14*), IQ (*t*(703) = -.21, *p = .82, d =.02*) or SRS (*t*(702) = .02, *p = .98, d = .002*).

***Additional measures***

In addition to the analysis of reaction times in relation to SRS scores we also analysed some other parameters derived from the eye-tracking data:

- **Accuracy:** it was defined as the number of right key pressings from the total number of valid trials.
- **Latency to first-fixation:** it was computed as the time that passed from the onset of the stimuli until the participant fixated for the first time the ‘target’ object. In order to calculate this value an Area of Interest (AOI) was defined around the ‘target’ object (see Figure 1). This AOI was calculated by expanding in all directions the size of the rectangular object using as parameter the minimum distance in pixels between the centre of the different objects in the trial. Since the minimum distance between objects changes depending on the set size, we choose as parameter the minimum distance of set size 28 in order to keep a constant value in all conditions.
- **Latency to key-press:** it was defined as the time that passed from the first fixation into the target until any key was-pressed.

**Supplementary Figures**


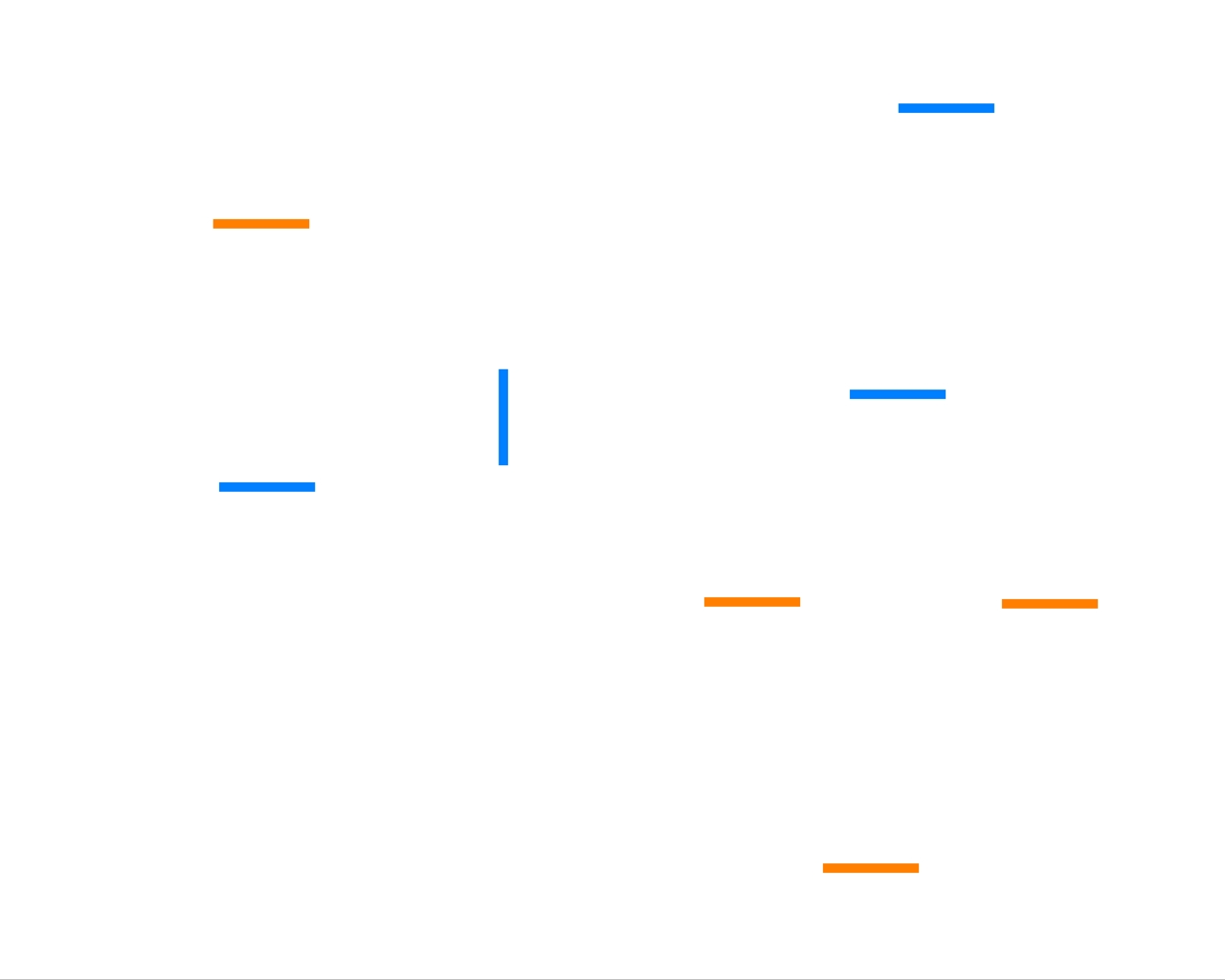

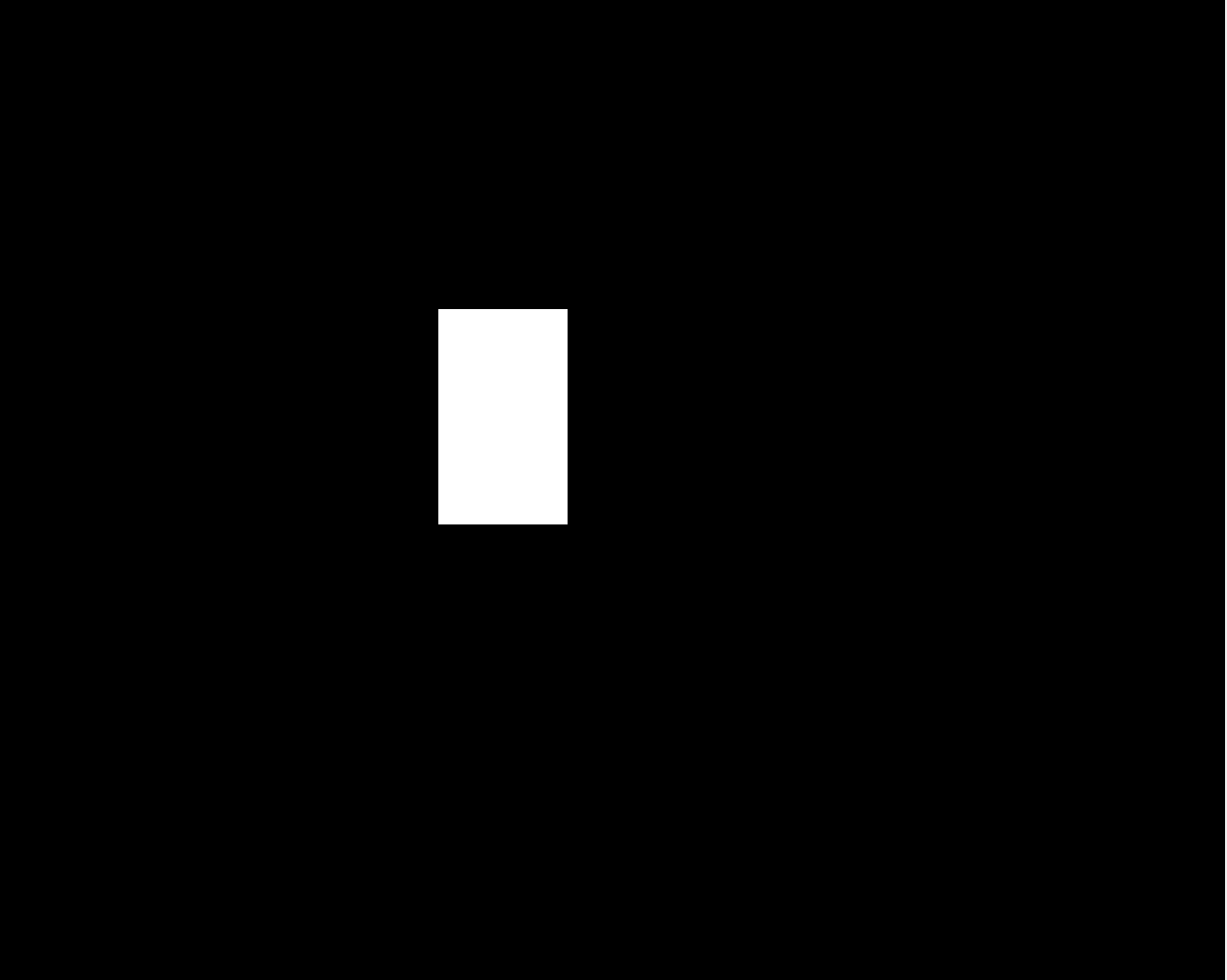


***Supplementary Figure 1****. Example of the definition area of interest (white rectangle on the right) used for the calculation of the latency variables. The target object (blue vertical rectangle) was expanded in all directions by a fixed amount of pixels.*

**
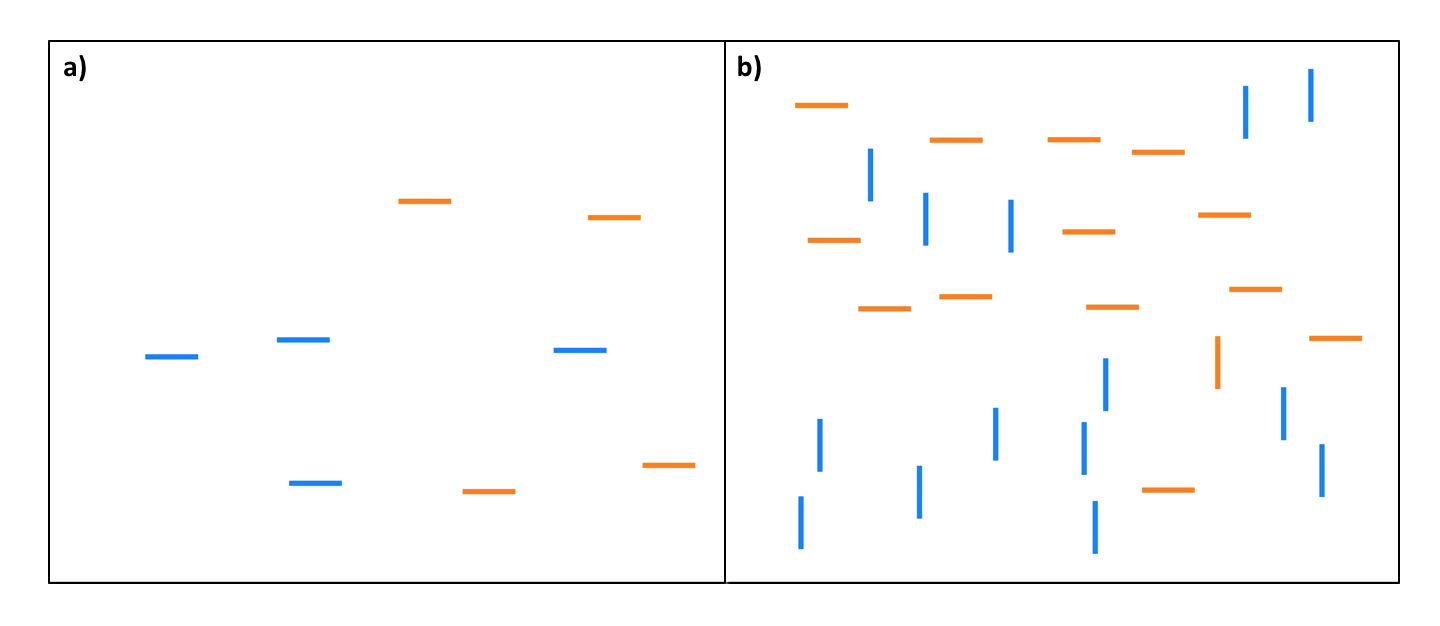
**

***Supplementary Figure 2****. Two examples of the stimuli presented during the visual search task, with an example of a feature absent (1a) and an example of the conjunctive target present trial (1b).*

*
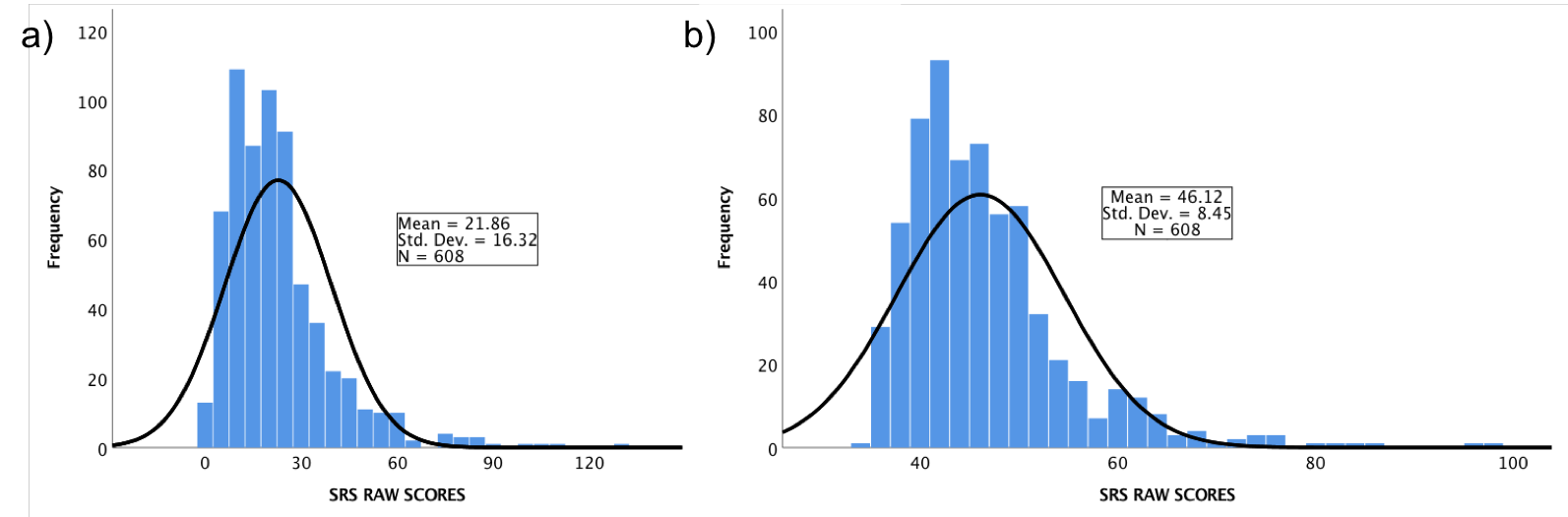
*

***Supplementary Figure 3****. Distribution of SRS scores. a) distribution of raw scores b) distribution of T scores. Although the SRS is not normed in Sweden, it is notable that the distribution is very similar to what has been observed in English speaking populations, with many individuals scoring above 65, a cut off for clinically significant ASD symptom levels (according to the SRS manual). We used raw scores for main analyses in this article, but results remained essentially unchanged if we used T-scores instead.*


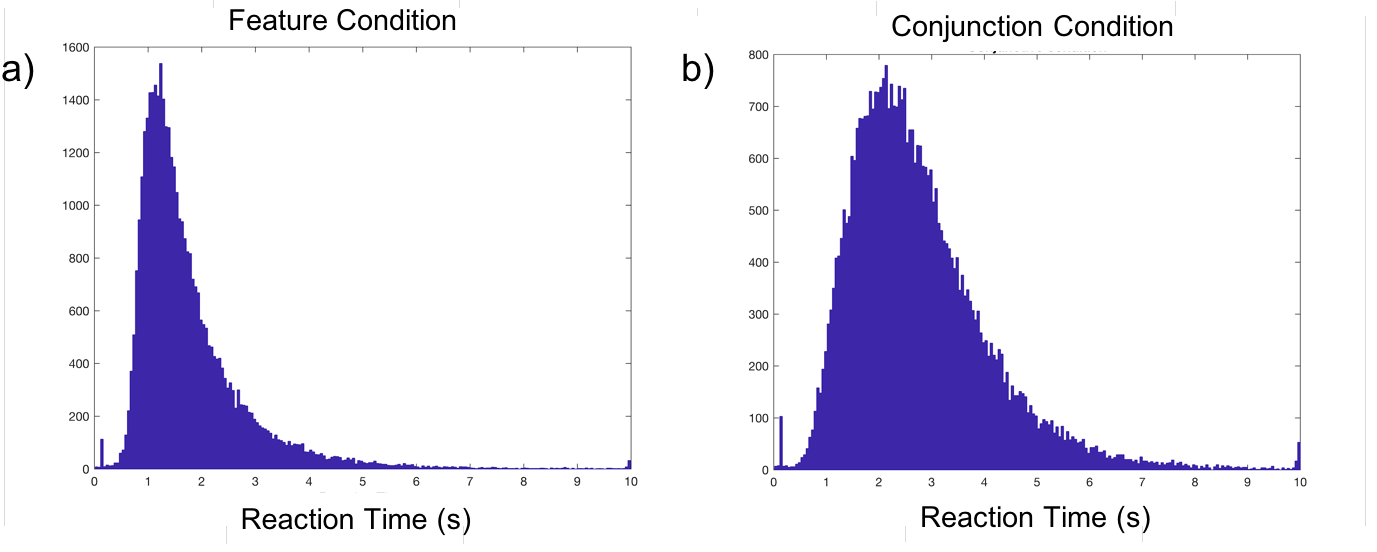


***Supplementary Figure 4.*** *Distributions of RTs (before any exclusions were applied) from individual trials for all participants. a) Distribution of RTs of feature condition b) Distribution of RTs of conjunctive condition.*

**Supplementary Tables**

***Supplementary Table 1.*** *Correlations between RTs and sex, age, overall IQ, IQ subscales, SRS and t-scaled SRS for all the individual conditions. The n varies slightly between analyses due to the availability of the RT measures.*

|  | **Feature 8 Absent** | **Feature 8 Present** | **Conjunction 8 Absent** | **Conjunction 8 Present** | **Feature 18 Absent** | **Feature 18 Present** | **Conjunction 18 Absent** | **Conjunction 18 Present** | **Feature 28 Absent** | **Feature 28 Present** | **Conjunction 28 Absent** | **Conjunction 28 Present** |
| --- | --- | --- | --- | --- | --- | --- | --- | --- | --- | --- | --- | --- |
| **RTs vs** **Sex** | r(603) = -.04 *p* = .29 | r(602) = -.03 *p* = .45 | r(605) = -.02 *p* = .60 | r(603) = .00  *p* = .88 | r(602) = -.05 *p* = .22 | r(597) = -.08 *p* = .038 | r(605) = -.00 *p* = .94 | r(601) = -.01  *p* = .69 | r(603) = -.05 *p* = .43 | r(600) = .03 *p* = .17 | r(605) = -.00 *p* = .96 | r(605) = -.01  *p* = .68 |
| **RTs vs** **Age** | r(602) = -.24 *p* < .001 | r(601) = -.25 *p* < .001 | r(605) = -.19 *p* < .001 | r(603) = -.19 *p* < .001 | r(602) = -.20 *p* < .001 | r(597) = -.25 *p* < .001 | r(605) = -.12 *p* < .01 | r(601) = -.21 *p* < .001 | r(603) = -.16 *p* < .001 | r(600) = -.24 *p* < .001 | r(604) = -.12  p = .003 | r(604) = -.16  p < .001 |
| **RTs vs IQ(all)** | r(605) = -.07 *p* = .07 | r(605) = -.18 *p* < .001 | r(585) = -.09 *p =* .02 | r(583) = -.16 *p* < .001 | r(582) = -.15  *p* < .001 | r(577) = -.14 *p* < .001 | r(585) = -.11 *p* < .01 | r(582) = -.11 *p* < .01 | r(583) = -.13 *p =* .001 | r(580) = -.15 *p* < .001 | r(559) = -.11  p = .006 | r(560) = -.15  p < .001 |
| **RTs vs IQ Coding** | r(558) = -.23 *p* < .001 | r(556) = -.22 *p* < .001 | r(579) = -.19 *p* < .001 | r(577) = -.19 *p* < .001 | r(576) = -.26 *p* < .001 | r(571) = -.25 *p* < .001 | r(579) = -.25 *p* < .001 | r(575) = -.18 *p* < .001 | r(577) = -.23 *p* < .001 | r(574) = -.21 *p* < .001 | r(559) = -.21  p < .001 | r(560) = -.21  p < .001 |
| **RTs vs IQ Matrices** | r(580) = -.01  *p* = .65 | r(579) = -.09  *p* = .02 | r(582) = -.03  *p* = .36 | r(580) = -.08  *p* = .04 | r(579) = -.07  *p* = .058 | r(574) = -.06  *p* = .09 | r(582) = -.04  *p* = .23 | r(578) = -.04  *p* = .28 | r(580) = -.09  *p* = .01 | r(577) = -.08  *p* = .03 | r(582) = -.01  *p* = .66 | r(582) = -.06  *p* = .11 |
| **RTs vs IQ Vocabulary** | r(574) = .08  *p* = .046 | r(572) = -.03  *p* = .39 | r(576) = .04  *p* = .30 | r(574) = -.06  *p* = .14 | r(572) = -.03  *p* = .40 | r(568) = -.01  *p* = .75 | r(575) = .04  *p* = .30 | r(572) = -.00  *p* = .91 | r(574) = .03  *p* = .40 | r(571) = .00  *p* = .92 | r(575) = -.00  *p* = .83 | r(576) = -.03  *p* = .36 |
| **RTs vs IQ Number_Rep** | r(575) = -.03  *p* = .39 | r(574) = -.09  *p* = .01 | r(577) = -.03  *p* = .34 | r(575) = -.07  *p* = .08 | r(574) = -.08  *p* = .05 | r(569) = -.04  *p* = .28 | r(577) = -.04  *p* = .32 | r(573) = -.05  *p* = .16 | r(575) = -.07  *p* = .07 | r(572) = -.10  *p* = .01 | r(577) = -.08  *p* = .04 | r(577) = -.07  *p* = .05 |
| **RTs vs SRS** | r(585) = .01 *p* = .79 | r(584) = -.04 *p* = .31 | r(584) = -.00 *p* = .90 | r(582) = .00 *p* = .93 | r(581) = .06 *p* = .10 | r(576) = -.03 *p* = .38 | r(584) = .00 *p* = .88 | r(580) = .00 *p* = .90 | r(582) = -.01 *p* = .93 | r(579) = -.01 *p* = .67 | r(584) = .00  p = .90 | r(584) = .00  p = .93 |
| **RTs vs** **Scaled-SRS** | r(582) = .00  *p* = .88 | r(583) = -.05  *p* = .21 | r(584) = -.00  *p* = .86 | r(582) = .00  *p* = .90 | r(581) = .06  *p* = .15 | r(576) = -.04 *p* = .26 | r(584) = .00  *p* = .92 | r(580) = .00  *p* = .92 | r(582) = -.01  *p* = .81 | r(579) = -.02  *p* = .51 | r(584) = .00  *p* = .95 | r(584) = .00  *p* = .98 |

***Supplementary Table 2.*** *Correlations between time to first fixation at target, gaze fixation vs key-press difference and accuracy and SRS scales. The X represent does trials without the target present in the trial, where the eye movement measures cannot be calculated. The n varies slightly between analyses due to the availability of the different measures.*

|  | **Feature 8 Absent** | **Feature 8 Present** | **Conjunction 8 Absent** | **Conjunction 8 Present** | **Feature 18 Absent** | **Feature 18 Present** | **Conjunction 18 Absent** | **Conjunction 18 Present** | **Feature 28 Absent** | **Feature 28 Present** | **Conjunction 28 Absent** | **Conjunction 28 Present** |
| --- | --- | --- | --- | --- | --- | --- | --- | --- | --- | --- | --- | --- |
| **First-Fixation vs SRS** | X | r(581) = -.03  *p* = .45 | X | r(581) = -.06  *p* = .15 | X | r(576) = -.01  *p* = .65 | X | r(580) = .00  *p* = .98 | X | r(579) = .04  *p* = .31 | X | r(584) = -.01  *p* = .77 |
| **Key-press diff vs SRS** | X | r(581) = -.03  *p* = .45 | X | r(582) = .05  *p* = .15 | X | r(576) = -.03  *p* = .41 | X | r(580) = .00  *p* = .87 | X | r(579) = -.04  *p* = .24 | X | r(584) = .01  *p* = .71 |
| **Accuracy vs SRS** | r(582) = -.00  *p* = .89 | r(581) = -.02  *p* = .55 | r(584) = -.05  *p* = .21 | r(582) = .01  *p* = .68 | r(581) = .01  *p* = .65 | r(576) = -.00  *p* = .96 | r(584) = -.00  *p* = .85 | r(580) = -.08  *p* = .055 | r(582) = -.01  *p* = .76 | r(579) = .03  *p* = .45 | r(584) = -.04  *p* = .28 | r(584) = -.02  *p* = .52 |

***Supplementary Table 3.*** *Correlations between SRS and visual search efficiency (intercepts and slopes for set size by reaction time function) defined in terms of time to first fixation at target, key-press latency vs gaze fixation latency difference score, and accuracy. The X represent does trials the eye movement measures cannot be calculated (Absent). The n varies slightly between analyses due to the availability of the different measures and the removal of outliers.*

|  | **All Conditions**  **Intercept** | **All Conditions**  **Slope** | **Present**  **Intercept** | **Present**  **Slope** | **Absent**  **Intercept** | **Absent**  **Slope** | **Conjunction Intercept** | **Conjunction Slope** | **Feature Intercept** | **Feature Slope** |
| --- | --- | --- | --- | --- | --- | --- | --- | --- | --- | --- |
| **First-Fixation vs SRS** | r(578) = -.05  *p* = .19 | r(578) = .07  *p* = .07 | r(578) = -.05  *p* = .19 | r(578) = .07  *p* = .07 | X | X | r(580) = -.03  *p* = .45 | r(580) = .03  *p* = .42 | r(573) = -.08  *p* = .047 | r(573) = .07  *p* = .06 |
| **Key-press diff vs SRS** | r(579) = -.03  *p* = .39 | r(579) = -.03  *p* = .48 | r(579) = -.03  *p* = .39 | r(579) = -.03  *p* = .48 | X | X | r(576) = .07  *p* = .06 | r(576) = -.05  *p* = .16 | r(579) = .02  *p* = .50 | r(579) = -.05  *p* = .19 |
| **Accuracy vs SRS** | r(572) = -.02  *p* = .57 | r(572) = .01  *p* = .73 | r(553) = .03  *p* = .48 | r(553) = .01  *p* = .73 | r(556) = -.08  *p* = .046 | r(556) = .06  *p* = .10 | r(567) = -.07  *p* = .06 | r(567) = .06  *p* = .13 | r(561) = -.01  *p* = .66 | r(561) = .04  *p* = .36 |

***Supplementary Table 4^1^.*** *Extreme group analysis. Partial correlations controlling for age between RTs and SRS scales for participants with SRS >= 55 and for participants with SRS < 55 for the remaining conditions.*

|  | **Feature 8 Absent** | **Feature 8 Present** | **Conjunction 8 Absent** | **Conjunction 8 Present** | **Feature 18 Absent** | **Feature 18 Present** | **Conjunction 18 Absent** | **Conjunction 18 Present** | **Feature 28 Absent** | **Feature 28 Present** |
| --- | --- | --- | --- | --- | --- | --- | --- | --- | --- | --- |
| **SRS >= 55** | r(28) = -.02  *p* = .91 | r(30) = -.15  *p* = .40 | r(29) = -.13  *p* = .48 | r(28) = -.01  *p* = .95 | r(29) = .05  *p* = .76 | r(25) = -.06  *p* = .76 | r(29) = -.08  *p* = .66 | r(27) = -.15  *p* = .43 | r(28) = -.20  *p* = .30 | r(27) = -.018  *p* = .92 |
| **SRS < 55** | r(557) = .01  *p* = .64 | r(554) = -.03  *p* = .46 | r(555) = .02  *p* = .59 | r(554) = .021  *p* = .72 | r(552) = -.00  *p* = .91 | r(551) = .00  *p* = .92 | r(555) = .02  *p* = .53 | r(553) = -.00  *p* = .94 | r(554) = .02  *p* = .55 | r(552) = .01  *p* = .69 |

***^1^Group difference analyses:*** Mann Whitney test showed no significant differences in any of the conditions (*U_conj_8_absent_* = 7205.5; *p*= .49, *d = -*.01; *U_conj_8_absent_* = 7520.5; *p*= .38, *d = -*.01; *U_conj_8_absent_* = 7722.0; *p*= .67, *d = -*.01; *U_conj_8_present_* = 7530.0; *p*= .75, *d = -*.01; *U_feat_18_absent_* = 7768.0; *p*= .75, *d = -*.01; *U_feat_18_present_* = 5198.0; *p*= .03, *d = -*.08; *U_conj_18_absent_* = 8039.5; *p*= .95, *d = -*.002; *U_conj_18_present_* = 7326.0; *p*= .83, *d = -*.008; *U_feat_28_absent_* = 6884.0; *p*= .29, *d = -*.04; *U_feat_28_present_* = 6111.0; *p*= .10, *d = -*.06).

***Supplementary Table 5^1^.*** *Extreme group analysis. Correlations between the visual search efficiency (intercepts and slopes for set size by reaction time function) and SRS scales for participants with SRS >= 55 and for participants with SRS < 55 for the remaining conditions.*

|  | **Present**  **Intercept** | **Present**  **Slope** | **Absent**  **Intercept** | **Absent**  **Slope** | **Conjunction Intercept** | **Conjunction Slope** | **Feature Intercept** | **Feature Slope** |
| --- | --- | --- | --- | --- | --- | --- | --- | --- |
| **SRS >= 55** | r(25) = -.16  *p* = .40 | r(25) = -.17  *p* = .37 | r(25) = .19  *p* = .33 | r(25) = -.30  *p* = .11 | r(25) = .03  *p* = .85 | r(25) = -.25  *p* = .18 | r(24) = .09  *p* = .65 | r(24) = -.04  *p* = .84 |
| **SRS < 55** | r(548) = -.23  *p* = .58 | r(548) = .03  *p* = .43 | r(552) = -.04  *p* = .32 | r(552) = .06  *p* = .11 | r(551) = -.02  *p* = .58 | r(551) = .04  *p* = .24 | r(551) = -.03  *p* = .46 | r(551) = .05  *p* = .23 |

***^1^Group difference analyses:*** Mann Whitney test showed no significant differences in any of the conditions (*U_present_interc_* = 8110.5; *p*= .81, *d =* .13; *U_present _slope_* = 7933.0; *p*= .92, *d =* .11; *U_absent_interc_* = 8090.5; *p*= .76, *d =* .12; *U_absent_slope_* = 8092.5; *p*= .75, *d =* .12; *U_conj_interc_* = 8268.0; *p*= .91, *d =* .14; *U _conj_slope_* = 8061.5; *p*= .74, *d =* .12; *U_feat_interc_* = 5020.0; *p*= .07, *d =* .16; *U_feat_slope_* = 5971.0; *p*= .36, *d =* .06).

***Supplementary Table 6*** *Sensitivity analyses. Correlations between mean RTs, median RTs, mean RTs using an upper cut-off +3SD, median RTs with anticipatory cut-off 150 ms and upper cut-off 3SD, mean RTs with all RT valid irrespectively of the validity of gaze data and t-scaled SRS scores (see main text). Additional correlations controlling for age and IQ were performed between RTs and SRS. The n varies slightly between analyses due to the availability of the different measures.*

|  | **Feature 8 Absent** | **Feature 8 Present** | **Conjunction 8 Absent** | **Conjunction 8 Present** | **Feature 18 Absent** | **Feature 18 Present** | **Conjunction 18 Absent** | **Conjunction 18 Present** | **Feature 28 Absent** | **Feature 28 Present** | **Conjunction 28 Absent** | **Conjunction**  **28 Present** |
| --- | --- | --- | --- | --- | --- | --- | --- | --- | --- | --- | --- | --- |
| **RTs vs SRS** | r(585) = .01 *p* = .79 | r(584) = -.04 *p* = .31 | r(584) = -.00 *p* = .90 | r(582) = .00 *p* = .93 | r(581) = .06 *p* = .10 | r(576) = -.03 *p* = .38 | r(584) = .00 *p* = .88 | r(580) = .00 *p* = .90 | r(582) = -.01 *p* = .93 | r(579) = -.01 *p* = .67 | r(584) = .00  p = .90 | r(584) = .00  p = .93 |
| **Median RTs vs SRS** | r(585) = .01 *p* = .73 | r(584) = -.01 *p* = .66 | r(584) = .01 *p* = .81 | r(582) = -.00 *p* = .87 | r(581) = .08 *p* = .04 | r(576) = .00 *p* = .99 | r(584) = .00 *p* = .83 | r(580) = .02 *p* = .57 | r(582) = .01 *p* = .72 | r(579) = -.00 *p* = .98 | r(584) = .00  p = .86 | r(584) = -.00  p = .82 |
| **RTs_3SD vs SRS** | r(585) = .01 *p* = .75 | r(584) = -.03 *p* = .34 | r(584) = -.00 *p* = .94 | r(582) = .00 *p* = .90 | r(581) = .06 *p* = .13 | r(576) = -.02 *p* = .63 | r(584) = -.00 *p* = .96 | r(580) = .00 *p* = .91 | r(582) = .00 *p* = .98 | r(579) = .01 *p* = .72 | r(584) = -.00  p = .94 | r(584) = .00  p = .82 |
| **MedianRTs_3SD vs SRS** | r(585) = .00 *p* = .83 | r(584) = -.03 *p* = .43 | r(584) = .00 *p* = .94 | r(582) = -.00 *p* = .87 | r(581) = .06 *p* = .13 | r(576) = -.00 *p* = .86 | r(584) = .00 *p* = .84 | r(580) = .02 *p* = .56 | r(582) = .00 *p* = .82 | r(579) = -.00 *p* = .96 | r(584) = -.00  p = .87 | r(584) = -.01  p = .80 |
| **RTs All Valid vs SRS** | r(585) = .00 *p* = .92 | r(584) = -.01 *p* = .64 | r(584) = .02 *p* = .49 | r(582) = .02 *p* = .49 | r(581) = .02 *p* = .55 | r(576) = .02 *p* = .62 | r(584) = -.01 *p* = .81 | r(580) = .01 *p* = .80 | r(582) = -.01 *p* = .70 | r(579) = .01 *p* = .79 | r(584) = -.01  p = .75 | r(584) = .02  p = .47 |
| **RTs vs SRS**  **(Age control)** | r(582) = .02  *p* = .55 | r(583) = -.03  *p* = .39 | r(584) = .00  *p* = .87 | r(582) = .02  *p* = .63 | r(581) = .07 *p* = .06 | r(576) = -.02 *p* = .49 | r(584) = .01  *p* = .78 | r(580) = .01  *p* = .68 | r(582) = .00  *p* = .95 | r(579) = -.00  *p* = .85 | r(584) = .01  *p* = .79 | r(584) = .01  *p* = .77 |
| **RTs vs SRS (IQ Control)** | r(580) = -.02 *p* = .48 | r(578) = -.03 *p* = .44 | r(578) = -.00 *p* = .89 | r(577) = -.08 *p* = .05 | r(580) = -.02 *p* = .60 | r(576) = -.02 *p* = .55 | r(577) = -.07 *p* = .48 | r(572) = -.02 *p* = .07 | r(580) = -.02 *p* = .54 | r(580) = -.03 *p* = .41 | r(578) = -.02  p = .51 | r(575) = -.05  p = .20 |
